# Migraine, obesity and body fat distribution – a population-based study

**DOI:** 10.1186/s10194-020-01163-w

**Published:** 2020-08-06

**Authors:** Espen Saxhaug Kristoffersen, Sigrid Børte, Knut Hagen, John-Anker Zwart, Bendik Slagsvold Winsvold

**Affiliations:** 1 Research and Communication Unit for Musculoskeletal Health, Division of Clinical Neuroscience, Oslo University Hospital, Oslo, Norway; 2 Department of General Practice, HELSAM, University of Oslo, PO Box 1130, Blindern, 0318 Oslo, Norway; 3 Department of Neurology, Akershus University Hospital, Lørenskog, Norway; 4 Department of Neuromedicine and Movement science, Norwegian University of Science and Technology, Trondheim, Norway; 5 Clinical Research Unit Central Norway, St. Olavs University Hospital, Trondheim, Norway; 6 Institute of Clinical Medicine, University of Oslo, Oslo, Norway; 7 Department of Research, Innovation and Education, Division of Clinical Neuroscience, Oslo University Hospital, Oslo, Norway

**Keywords:** Body mass index, Obese, Pain, General population

## Abstract

**Background:**

Obesity has been linked to an increased prevalence of migraine, and to increased migraine attack frequency, but several questions are left unanswered by previous studies. We examined the relationship between obesity and headache in a large, population-based study where we could take into account body fat distribution, migraine subtypes and tension-type headache.

**Methods:**

The third population-based Nord-Trøndelag Health Study (HUNT3) included validated headache questionnaires and objective anthropometric measurements. Using a cross-sectional design, our sample consisted of 18,191 women and 14,985 men, aged 19 to 96 years. Of these 4290 (12.9%) had migraine, 4447 (13.4%) had frequent tension-type headache (TTH), and 24,439 were headache-free controls. A total of 5049 individuals with unclassified headache were excluded from the analyses. Using logistic regression, we modeled the association between obesity and headache prevalence, adjusting for relevant confounders.

**Results:**

Both total body obesity (TBO) and abdominal obesity (AO) were associated with a higher prevalence of migraine when compared to headache-free controls (OR 1.45 95% CI 1.32–1.59 and OR 1.29 95% CI 1.18–1.41, respectively), in particular for individuals < 50 years of age (OR 1.74 95% CI 1.54–1.98 and OR 1.89 95% CI 1.69–2.11). Similar results were seen for migraine with and without aura. Similar Overall, a weaker associations were as observed between obesity and TTH. There was a dose-response relationship between obesity categories and increased headache frequency in subjects with migraine. TBO was associated with migraine prevalence and attack frequency independent of AO.

**Conclusion:**

Both TBO and AO were associated with migraine prevalence and attack frequency. This association was largely limited to individuals < 50 years of age. TBO, rather than AO, may be a better measure of obesity in relation to migraine.

## Background

The prevalence of overweight and obesity has increased substantially in the last decades, and is now one of the leading risk factors for disease and death worldwide [1, 2]. Total body obesity (TBO), as measured by body mass index (BMI), has been associated with migraine prevalence, and with the progression from episodic to chronic migraine [3–10]. Several questions are, however, left unanswered by previous studies. First, obesity defined from BMI cannot distinguish between fat and muscle mass or between abdominal and peripheral fat distribution [11, 12]. Abdominal visceral fat is metabolically different from other body fat, and appears to be an independent risk factor for medical complications [12, 13]. Abdominal obesity (AO) may be of particularly interest in migraine, as this adipose tissue produces multiple substances potentially involved in migraine pathophysiology, including markers of systemic inflammation [14]. Waist circumference has been suggested as a better measure of abdominal fat than BMI, and may be better suited to predict future health risks [11, 12, 15, 16]. However, most previous population-studies of obesity and migraine have been based on BMI rather than AO [11–13, 15]. Second, both fat distribution and migraine prevalence vary substantially with sex and age, and it is possible that the relationship between the two also changes with these factors [5, 8, 10, 17]. Interestingly, population-based studies of older individuals typically find no association between obesity and migraine, while studies of reproductive-age individuals find substantial associations [7, 8]. Third, tension-type headache (TTH) has typically not been examined by these population-based studies.

Our aim was to determine how obesity and body fat distribution TBO and AO were associated with migraine and TTH in a large population-based study. That included validated headache diagnoses and objective anthropomorphic measurements.

Secondly, we explored how this association changed with age and sex, and whether there was a dose-response relationship between obesity measures and headache frequency.

## Methods

### Study sample

All inhabitants 20 years or older in Nord-Trøndelag county of Norway were invited to participate in the third Nord-Trøndelag Health Study (HUNT3) (2006–2008). The study population, including both participants and non-participants has been described in detail previously [18]. In brief, two questionnaires including more than 200 health-related questions were given to the participants. Of the 94,194 individuals invited, 50,807 (54%) answered the first questionnaire (Q1) which was enclosed with the invitation letter. They were also invited to participate in a brief medical examination, including anthropometric measurements, and to fill in a second questionnaire (Q2), which included a total of 14 headache questions. In total, 38,225 (41%) participants answered Q2, had complete information on age, sex, smoking, depression measured by Hospital Anxiety and Depression Scale (HADS-D) and valid information regarding diabetes, and could be classified according to headache status. Of these 33,176 participants had either migraine, TTH or no headache, according to the classification described below, and were eligible for inclusion in the study, while 5049 participants with non-classifiable headache were excluded. There was complete information on BMI and waist circumference for 33,067 (99.7%) and 33,027 (99.6%).

### Headache classification

The 14 headache questions in the second questionnaire (Q2) were designed mainly to determine whether the individual had headache, the frequency of headache, and, when headache were reported, to diagnose migraine and TTH according to slight modified the second version of the International Classification of Headache Disorders (ICHD-II) [18, 19]. Subjects who answered “yes” to the screening question “Have you suffered from headache during the last 12 months?” were classified as headache sufferers. Those who answered “no” comprise the headache-free control group. Based on the subsequent headache questions, headache sufferers were classified as having migraine if they fulfilled the following three criteria: (1) headache attacks lasting ≤72 h, (< 4 h was accepted because participants were not asked for duration of untreated attacks in the question “How long does the headache usually last?); (2) headache had usually at least two of the following characteristics: pulsating quality, unilateral location, moderate/severe pain intensity or aggravation by physical activity; (3) during headache, at least one of the following: nausea/vomiting, photophobia and phonophobia. Migraine with aura was defined as migraine and typical visual disturbance prior or during headache. Headache sufferers were classified as having TTH if they fulfilled the following criteria: (i) Headache at least one day a month. (ii) Headache with at least two of the following characteristics: bilateral location, pressing quality, mild to moderate intensity and no aggravation by physical activity. (iii) During headache no nausea or vomiting and no phonophobia or photophobia. The headache diagnoses were mutually exclusive, with migraine taking precedence over TTH. Migraine or TTH was classified as episodic if the participant reported headache on ≤14 days per month, and chronic if > 14 days per month. The headache diagnoses have previously been validated against clinical interviews by neurologists [18]. The sensitivity and specificity were, respectively, 88% and 86% for any headache (Cohen’s kappa (κ) = 0.70, 95% CI 0.61–0.79), 51% and 95% for migraine (κ = 0.50, 95% CI 0.32–0.68), 50% and 95% for migraine with aura (κ = 0.44, 95% CI 0.38–0.50), and 96% and 69% for TTH (κ = 0.44, 95% CI 0.30–0.58).

### Anthropometric measurements

Height and weight were measured with standardized weight scales and metric bands. The participants wore light clothes and no shoes. Height was measured to the nearest 1.0 cm, and weight to the nearest 0.5 kg. BMI was defined as weight/height2 and computed in kg/m2. BMI status in adults were categorized into four TBO levels; underweight (BMI < 18.5), normal weight (18.5 ≤ BMI < 25), overweight (25 ≤ BMI < 30) and obese (BMI ≥ 30) in accordance to World Health Organization (WHO) definitions [11].

Waist circumference (WC) was measured to the nearest cm applying non-stretchable band horizontally at the umbilical level after the participants emptied their lungs, or midway between the last rib and the iliac cristae if the latter was larger. AO was defined from WC and categorized into three levels according to WHO criteria [11]; normal weight (women < 80 cm, men < 94 cm), abdominal overweight (women 80–88 cm, men 94–102 cm) and abdominal obesity (women > 88 cm, men > 102).

### Potential confounders

In accordance with a pre-planned strategy, it was decided to include the following variables available from HUNT3 as potential confounders because of their known impact on headache and obesity: sex (binary); age at participation (continuous); any history of diabetes (binary); current daily tobacco smoking (binary); and HADS-D (continuous). While HUNT3 included a wide range of health-related information, including several factors that have previously been associated with either headache or migraine [20], we limited the list to these potential confounders to avoid over-adjustment bias [21].

### Statistical analysis

The association between headache and obesity was tested using logistic regression models. We estimated odds ratios (OR) and 95% confidence intervals (CI) for the association between anthropometric measures (exposure) and headache (outcome), using headache-free participants as controls. The exposure was either TBO categories (4 levels) or abdominal obesity (3 levels) as described above. Separate analyses were performed for migraine and TTH. Based on previous knowledge that body fat distribution and headache prevalence differ strongly by sex and age, we next performed analyses stratified by sex and by age < 50 years or ≥ 50 years of age. The cut-off of 50 years was based on the prior knowledge of headache prevalence and the hormonal changes in women during the menopause [22]. Lastly, to estimate whether the effects of TBO and abdominal obesity were independent of each other, we modelled the effect of each, adjusting for the other. To limit the number of tests, this additional adjustment was included only in the main (non-stratified) analyses and in analyses of headache frequency. Two-tailed *P*-values are reported, using 5% as a cut-off for statistical significance. Data analysis was performed with the IBM Statistical Package for the Social Sciences, version 26 (SPSS, Chicago, Illinois, USA).

## Results

Our final sample consisted of 33,176 individuals, with a mean age of 54.4 years (range 19 to 96 years). Of these, 4290 (12.9%) had migraine and 4447 (13.4%) had TTH. Details are given in Table 1.

**Table 1:** Clinical profile of participants

	No headache *n* = 24,439	Migraine *n* = 4290	Tension-type headache *n* = 4447
*Discrete variables, n (%)*			
Female	12,280 (50.2)	3158 (73.6)	2753 (61.9)
Daily smoking	4237 (17.3)	933 (21.7)	844 (19.0)
Diabetes	1262 (5.2)	105 (2.4)	147 (3.3)
*Continuous variables, mean (SD)*			
Age (years)	56.7 (15.6)	46.2 (12.9)	49.3 (14.0)
HADS depression score	3.2 (2.8)	3.6 (3.2)	3.9 (2.9)
Body-mass index	27.2 (4.2)	27.3 (5.0)	27.3 (4.6)
Waist circumference	94.1 (11.9)	92.1 (13.2)	93.2 (12.8)

*SD* standard deviation, *HADS* Hospital Anxiety and Depression Scale

### Total body obesity

In adjusted analyses (Tables 2 and 3), TBO (BMI ≥ 30) was associated with increased odds for having migraine (OR 1.45, 95% CI 1.32–1.59), both for women and for men. The effect size was larger when considering only participants < 50 years of age (OR 1.74, 95% CI 1.54–1.98), and not seen for those ≥50 years. A more moderate association was observed in women between migraine and being overweight (BMI 25–30) (OR 1.23, 95% CI 1.11–1.35), not statistically significant in men (Table 3). In analyses of migraine subtypes, obesity was associated with higher odds for having both migraine with aura (OR 1.51, 95% CI 1.33–1.71) and migraine without aura (OR 1.43 95% CI 1.26–1.62). Likewise, being overweight (BMI 25–30) was associated with higher odds for having migraine with aura (OR 1.25 95% CI 1.12–1.39) and migraine without aura (OR 1.15 95% CI 1.04–1.29). Similar, but weaker associations were seen for TTH (Tables 2 and 3). In supplementary analyses, When including additionally adjustmented for degree of abdominal obesity (3 levels), the association between migraine and obesity (OR 1.44, 95% CI 1.26–1.63) and migraine and overweight (OR 1.19 95% CI 1.08–1.31) remained, while the association between TTH and obesity (1.13 95% CI 1.00–1.28) and TTH and overweight (1.05 95% CI 0.96–1.15), were less clear (data not shown). weaker.

**Table 2:** Association between obesity and headache

	No headache *N*	Migraine OR (95% CI) *N*	Tension-type headache OR (95% CI) *N*
**Obesity (BMI)**^a^			
Underweight	121	1.17 (0.78–1.74) 38	0.83 (0.53–1.31) 24
Normal weight	7519	1.0 (Ref.) 1455	1.0 (Ref.) 1469
Overweight	11,289	1.19*** (1.10–1.29) 1710	1.11** (1.03–1.20) 1893
Obese	5424	1.45*** (1.32–1.59) 1071	1.26*** (1.15–1.38) 1054
**Abdominal obesity (WC)**^b^			
Normal weight	6633	1.0 (Ref.) 1090	1.0 (Ref.) 1141
Abdominal overweight	7638	1.16** (1.05–1.27) 1232	1.20*** (1.09–1.31) 1367
Abdominal obesity	10,066	1.29*** (1.18–1.41) 1935	1.27*** (1.17–1.39) 1925

**p < 0.01; ***p < 0.001

^a^Underweight (BMI < 18.5), normal weight (18.5 ≤ BMI < 25), overweight (25 ≤ BMI < 30), obese (BMI ≥ 30)

^b^Normal weight (WC for women < 80 cm, for men < 94 cm), abdominal overweight (women 80–88 cm, men 94–102 cm), abdominal obesity (women > 88 cm, men > 102)

*OR* odds ratio, *CI* confidence intervals, *Ref*. reference category, *BMI* body mass index, *WC* waist circumference

The analyses are adjusted for age, sex, smoking, depression (HADS-D) and presence of diabetes

**Table 3:** Association between BMI and headache, stratified analyses

	Women			Men			Age < 50 years			Age ≥ 50 years		
	No headache N	Migraine OR (95% CI) N	Tension-type headache OR (95% CI) N	No headache N	Migraine OR (95% CI) N	Tension-type headache OR (95% CI) N	No headache N	Migraine OR (95% CI) N	Tension-type headache OR (95% CI) N	No headache N	Migraine OR (95% CI) N	Tension-type headache OR (95% CI) N
**Obesity (BMI)**†												
Underweight	92	1.12 (0.72–1.74) 32	0.81 (0.49–1.34) 20	29	1.36 (0.52–3.56) 6	0.81 (0.27–2.40) 4	53	1.33 (0.83–2.16) 29	0.80 (0.44–1.45) 14	68	1.51 (0.73–3.12) 9	1.27 (0.64–2.51) 10
Normal weight	4509	1.0 (Ref.) 1196	1.0 (Ref.) 1101	3010	1.0 (Ref.) 259	1.0 (Ref.) 368	3148	1.0 (Ref.) 964	1.0 (Ref.) 887	4371	1.0 (Ref.) 491	1.0 (Ref.) 582
Overweight	4794	1.23*** (1.11–1.35) 1163	1.06 (0.96–1.17) 995	6495	1.17 (1.00–1.37) 547	1.25** (1.09–1.42) 898	3241	1.24*** (1.11–1.38) 969	1.13* (1.10–1.26) 892	8048	0.96 (0.85–1.09) 741	0.99 (0.88–1.10) 1001
Obese	2841	1.38*** (1.24–1.54) 755	1.18** (1.05–1.32) 632	2583	1.65*** (1.38–1.98) 316	1.46*** (1.25–1.70) 422	1371	1.74*** (1.54–1.98) 650	1.45*** (1.28–1.66) 525	4053	1.00 (0.87–1.16) 421	1.00 (0.88–1.14) 529
**Abdominal obesity (WC)** †												
Normal weight	2306	1.0 (Ref.) 693	1.0 (Ref.) 585	4327	1.0 (Ref.) 397	1.0 (Ref.) 556	2969	1.0 (Ref.) 743	1.0 (Ref.) 697	3664	1.0 (Ref.) 347	1.0 (Ref.) 444
Abdominal overweight	3296	1.08 (0.95–1.21) 840	1.09 (0.96–1.23) 759	4342	1.25** (1.08–1.46) 392	1.28*** (1.13–1.45) 608	2397	1.25*** (1.11–1.41) 729	1.25*** (1.11–1.41) 695	5241	1.05 (0.91–1.22) 503	1.08 (0.95–1.23) 672
Abdominal obesity	6898	1.22*** (1.09–1.36) 1595	1.15* (1.03–1.29) 1396	3468	1.41*** (1.20–1.65) 340	1.44*** (1.26–1.65) 529	2400	1.89*** (1.69–2.11) 1121	1.62*** (1.45–1.82) 917	7666	1.26** (1.10–1.45) 814	1.17* (1.04–1.32) 1008

*p < 0.05; **p < 0.01; ***p < 0.001

†Underweight (BMI < 18.5), normal weight (18.5 ≤ BMI < 25), overweight (25 ≤ BMI < 30), obese (BMI ≥ 30)

†Normal weight (WC for women < 80 cm, for men < 94 cm), abdominal overweight (women 80–88 cm, men 94–102 cm), abdominal obesity (women > 88 cm, men > 102)

*OR* odds ratio, *CI* confidence intervals, *Ref*. reference category, *BMI* body mass index, *WC* waist circumference

The analyses are adjusted for age, sex, smoking, depression (HADS-D) and presence of diabetes

### Abdominal obesity (AO)

Results for AO in adjusted analyses (Tables 2 and 3) were similar to those for BMI. AB (WC > 88 cm in women, > 102 cm in men) was associated with increased odds for having migraine (OR 1.29, 95% CI 1.18–1.41), seen both for women and for men. The effect size was larger when considering only participants < 50 years (OR 1.89, 95% CI 1.69–2.11), but there was also an association for participants ≥50 years of age (OR 1.26, 95% CI 1.10–1.45). A more moderate association was observed between migraine and being abdominal overweight (WC 80–88 in women, 94–102 in men), not statistically significant in women. In analyses of migraine subtypes, abdominal obesity was associated with higher odds for having both migraine with aura (OR 1.39 95% CI 1.23–1.57) and migraine without aura (OR 1.21 95% CI 1.07–1.36). Likewise, being abdominal overweight was associated with higher odds for having migraine with aura (OR 1.15 95% CI 1.01–1.31) and migraine without aura (OR 1.16 95% CI 1.02–1.31). Associations between TTH and abdominal overweight and obesity were similar to those seen for migraine (Tables 2 and 3). In supplementary analyses, including adjustment When additionally adjusted for degree of TBO (4 levels), there was no association between migraine and AO (OR 1.02, 95% CI 0.90–1.16) or between migraine and abdominal overweight (OR 1.06 95% CI 0.96–1.17), while for TTH there was still an association with AO (OR 1.16 95% CI 1.03–1.30) and abdominal overweight (OR 1.15 95% CI 1.05–1.27) (data not shown).

### Obesity and headache frequency

TBO (BMI ≥ 30) and AO (WC > 88 cm in women, > 102 cm in men) were associated with both episodic and chronic migraine, with higher effect sizes for chronic than for episodic migraine (Table 4). TBO and AO were associated with episodic, but not with chronic, TTH.

**Table 4:** Obesity by headache frequency

		Non-obese (BMI) A	Obesity (BMI) B	Non-obese (WC) C	Abdominal obesity (WC)D
**Headache status**	Headache frequency	N	OR (95% CI) N	N	OR (95% CI)
**No headache**		18,929	1.0 (Ref.) 5424	14,271	1.0 (Ref.) 10,066
**Tension-type headache**	Episodic (≤14 days/month)	3141	1.15** (1.06–1.25) 964	2320	1.15*** (1.07–1.24) 1778
	Chronic (> 14 days/month)	245	1.27 (0.99–1.62) 90	188	1.03 (0.82–1.30) 147
**Migraine**	Episodic (≤14 days/month)	3015	1.25*** (1.16–1.36) 995	2191	1.15*** (1.07–1.24) 1805
	Chronic (> 14 days/month)	174	1.53** (1.16–2.02) 74	120	1.36* (1.04–1.76) S125

*OR* odds ratio, *CI* confidence intervals, *Ref.* reference category, *BMI* body mass index

**A**Body-mass index < 30. **B**Body-mass index ≥30. **C**Waist circumference ≤ 88 cm for women, ≤102 cm for men. **D**Waist circumference > 88 cm for women, > 102 cm for men. *p < 0.05; **p < 0.01; ***p < 0.001. Analyses are adjusted for age, sex, smoking, depression (HADS-D) and presence of diabetes

For subjects with migraine, the association between headache frequency and TBO remained after additionally adjusting the model for AO (2 levels; obese or non-obese) (OR 1.19 95% CI 1.08–1.32 for episodic headache; OR 1.43, 95% CI 1.02–2.00 for chronic headache). In contrast, after adjusting for TBO (2 levels; obese or non-obese), there was no remaining association between headache frequency and AO (OR 1.04 95% CI 0.95–1.14 for episodic headache; OR 1.10 95% CI 0.80–1.51 for chronic headache). In corresponding adjusted analyses for TTH, only a weak association remained between episodic TTH and AO, after adjusting for TBO (OR 1.10 95% CI 1.01–1.20).

## Discussion

In this large population-based study both TBO and AO were associated with a higher prevalence of migraine. The associations were mostly limited to individuals < 50 years of age. There was a dose-response relationship between obesity categories and increased headache frequency in subjects with migraine. Regarding body fat distribution, the association between migraine and TBO was independent of AO, but not vice versa, suggesting that TBO may be a more important measure with regards to migraine prevalence and chronification. Similar, but weaker associations, were seen for TTH.

The relationship between headaches and obesity was suggested in a prospective population-based 11-months follow-up study from US in 2003 [6]. Study subjects with increased BMI (> 25) had an increased relative risk of developing chronic headache (> 14 days/months), compared to normal-weight individuals. This study did not differentiate between headache types, but other cross-sectional population-based studies found an association between obesity and increased migraine frequency, giving credibility to the notion that obesity is a driving factor for migraine progression [3, 4, 9, 23]. In line with previous studies, we found a dose-response relationship between obesity and migraine frequency. Our results also indicate that the association is driven by TBO rather than by AO.

Obesity has also been associated with an increased prevalence of migraine. Two recent meta-analyses found that obese individuals had an overall increased risk of migraine of 14% versus 27% when compared with those with normal weight [7, 8]. However, most of the studies reviewed were based on BMI from self-reported height and weight. Also, the authors commented that further studies of the association between migraine and obesity should take into account age, gender, headache type and headache frequency, to provide more robust evidence. Ageing is associated with a change in the ratio of fat and lean body mass even in those with an unchanged BMI. In addition, the overall adipose tissue distribution is different in women and men, with younger women having more adipose tissue in a gluteofemoral distribution than abdominally, while men of all ages and older women have more abdominal adipose tissue depots then young women. One, previous population-based study examined the prevalence of migraine by TBO and AO, and found that the association between migraine and obesity varied by age, sex, and adipose tissue distribution [5]. In men and women aged 55 years or younger, migraine prevalence was increased in those with TBO (BMI ≥ 30), independent of AO. In men older than 55 years, migraine was not associated with obesity. However, in women older than 55 years, migraine prevalence was decreased among those with AO. This study could not classify TTH or migraine with and without aura, and did not include measures of headache frequency [5]. Our results confirm this and previous reports of a positive association between migraine and both TBO and AO. We extend the results in showing a similar association for migraine with aura, migraine without aura, and TTH. We also confirm that the association between obesity and headache is mostly restricted to younger individuals.

Based on findings in pre-clinical and clinical studies various mechanisms have been suggested to explain the association between migraine and obesity, including metabolic and hormonal activity of adipose tissue, increased release of pro-inflammatory substances, neuroinflammation and neuropeptides involving hypothalamic function [7, 8, 17, 24, 25]. Adiponectin and leptin are adipokines that are mostly released from subcutaneous adipose tissue, and that may have nociceptive traits in themselves [17, 26–28]. Both adiponectin and leptin are increased in migraineurs between attacks, although they may be decreased during attacks [17, 29]. Leptin levels are also increased in correlation with pro-inflammatory cytokines IL-6 and TNF-a [29], which have also been found to be elevated in people with migraine [30]. Serotonin and orexin A are neuropeptides that function as appetite regulators and are linked to obesity. They are also possibly linked to migraine pathophysiology, however this is still debated, along with the involvement and dysregulation of hypothalamic and homeostatic pathways [31–33].

A causal relationship between migraine and obesity can have important implications for clinicians, patients and future public health strategies, also in light of the increased risk of cardiovascular diseases in both migraine and obesity [2, 16, 34, 35]. Studies that have investigated the effect of weight loss on migraine have mostly examined dietary interventions, but gastric bypass has also been evaluated [24]. A recent systematic review concludes that weight reduction improves the frequency and duration of attacks in patients who have migraine and obesity, independently of the type of intervention and the amount of weight reduction [36]. Follow-up studies are needed to confirm this, and to provide a better understanding on the mechanisms linking migraine and obesity.

Strengths of our study include the large and unselected population, and the use of validated headache diagnoses and standardized anthropometric measurements. The general health focus of the questionnaires decreases the risk of a specific selection bias in relation to headache diagnoses. In the multivariate analyses we could adjust for several relevant potential confounders. However, the possibility of residual confounding cannot be excluded in this observational study. The migraine diagnoses were validated and based on the ICHD-criteria [18]. Although HUNT3 used the ICHD-II criteria, the migraine criteria did not change to the present ICHD-3 [19, 37]. A limitation of the study was the use of questionnaire-based headache diagnoses rather than clinical interview. This will lead to a degree of misclassification between migraine and other types of headache. Furthermore, it should be highlighted that mutually exclusive headache diagnosis was set only in individuals answering “yes” to the screening question “Have you suffered from headache during the last 12 months?” This is the main reason of the very low prevalence of THH of 13.4% in the present study. Thus, individuals without headache also included those with infrequent TTH (or migraine) without defining themselves as being headache sufferers. Another limitation of the study is that our question-based headache diagnosis did not identify secondary headaches associated with obesity, e.g. idiopathic intracranial hypertension or obstructive sleep apnea [38, 39]. On the other hand, such secondary headaches are relatively rare in the general population [39, 40]. However, the agreement between questionnaire-based diagnosis of migraine and interview has previously been found to be acceptable [18]. Finally, it should also be highlighted that the cross-sectional design does not permit any conclusions about causality, and that the results should be generalized with caution based on the 42% responder rate to the HUNT3 headache questionnaire.

## Conclusion

In this large population-based study both total body obesity and abdominal obesity were associated with a higher prevalence of migraine. There was a dose-response relationship between increased obesity and increased headache frequency in subjects with migraine. These findings may have clinical implications for treatment of migraine and should be further investigated in population-based follow-up studies.

## Data Availability

The authors declare that the data supporting the findings of this study are available within the article.

## Abbreviations

HADS: Hospital Anxiety and Depression Scale
HUNT: The Nord-Trøndelag Health Study
ICHD: International Classification of Headache Disorders
CI: Confidence interval
BMI: Body Mass Index
WC: Waist Circumference
TBO: Total body obesity
AO: Abdominal obesity
OR: Odds Ratio
TTH: Tension-type headache
